# Simulations of Switchback, Fragmentation and Sunspot Pair in *δ*-Sunspots during Magnetic Flux Emergence

**DOI:** 10.3390/s21020586

**Published:** 2021-01-15

**Authors:** Che-Jui Chang, Jean-Fu Kiang

**Affiliations:** Graduate Institute of Communication Engineering, National Taiwan University, Taipei 10617, Taiwan; f06942024@ntu.edu.tw

**Keywords:** *δ*-sunspot, polarity inversion line (PIL), solar flare, coronal mass ejection (CME), magnetic flux emergence

## Abstract

Strong flares and coronal mass ejections (CMEs), launched from δ-sunspots, are the most catastrophic energy-releasing events in the solar system. The formations of δ-sunspots and relevant polarity inversion lines (PILs) are crucial for the understanding of flare eruptions and CMEs. In this work, the kink-stable, spot-spot-type δ-sunspots induced by flux emergence are simulated, under different subphotospheric initial conditions of magnetic field strength, radius, twist, and depth. The time evolution of various plasma variables of the δ-sunspots are simulated and compared with the observation data, including magnetic bipolar structures, relevant PILs, and temperature. The simulation results show that magnetic polarities display switchbacks at a certain stage and then split into numerous fragments. The simulated fragmentation phenomenon in some δ-sunspots may provide leads for future observations in the field.

## 1. Introduction

Plasma features in solar corona are commonly observed with X-ray and EUV instruments [1,2]. It was observed that active regions (ARs) are highly correlated with solar flares and coronal mass ejections (CMEs) [3]. Strong flares and CMEs, the most catastrophic energy-releasing events in the solar system, take place in δ-sunspots, which are complex ARs featuring sheared polarity inversion lines (PILs) [4]. Numerous powerful flare eruptions have been observed in δ-sunspots [5]. More than 80% of X-class flares observed with the Geostationary Operational Environmental Satellite (GOES) occurred in δ-sunspots [6,7].

The formations of δ-sunspots and associated PILs, containing sheared magnetic structures, are crucial to understand flare eruptions and CMEs. In a typical δ-sunspot, the umbrae of two opposite polarities share a common penumbra in white light observations [3]. The temperatures at the δ-sunspots are about 3000–4500 K.

In [8], the evolution of the flare-productive NOAA (National Oceanic and Atmospheric Administration) Active Region 9026 was studied. The emergence and decay of δ-sunspots were investigated. Many changes in sunspot structures were observed simultaneously during the decaying process. Two opposite magnetic polarities and a PIL appeared on the solar surface. A switchback was observed in the magnetogram during the decay of a central δ-sunspot. Each pair of emerging bipolar sunspots was originally connected by a magnetic flux tube before its emergence. Rapid motions started in both sides of the central δ-sunspot before a strong flare activity, followed by the collapse and disintegration of the central δ-sunspot. In [9], a decaying follower sunspot, NOAA AR 10773, was studied by using spectroscopic observations in white light from 7–12 June 2005. The umbra and penumbra shrank in size and became faint during the decaying process, with the umbra finally contracted to a pore.

The magnetic flux emerged from the solar interior to the overlying atmosphere to form an active region, accompanied by various explosive phenomena [10]. In [11], a 3D MHDmodel was applied to simulate the emergence of a twisted, Ω-shaped magnetic flux tube from the top layer of the solar convection zone into the atmosphere and the corona. The tube initially located in the convection zone took an Ω shape and expanded to the stably stratified atmosphere, driven by magnetic buoyancy instability, also known as Parker instability. The magnetic flux tube rose to the photosphere and formed a bipolar structure on the solar surface.

In [12], the emergence of a magnetic flux tube from the convection zone to the solar atmosphere was simulated by using a 3D MHD model. The magnetic flux was shown to erupt into an unmagnetized corona and a corona with a pre-existing ambient magnetic field, respectively. The buoyancy raised the magnetic flux tube to the photosphere in the first expansion, then into the solar atmosphere in the second expansion.

In [13], a subphotospheric magnetic flux tube was simulated to rise from the convection zone, then erupt in the form of a magnetic flux rope into the corona. Both the field-free corona and the corona with pre-existing magnetic field were studied. The relative contact angle between the interacting fields and the field strength were crucial parameters to the evolution of the eruption.

In [14], a 3D MHD model was applied to simulate a hot and fast coronal jet, which was followed by several eruptions induced by the emergence of a magnetic flux tube from the convection zone to a magnetized coronal hole. The shape and internal structure in different emergence phases of the magnetic flux tube were investigated. An emerging magnetically dominated plasma dome was surmounted by a current sheet, beneath which a hot and fast coronal jet was produced in an inverted-Y shape.

In [15], emerging magnetic flux tubes with different initial magnetic field strengths, radii, twists, and lengths were simulated by using a 3D MHD model to investigate the effects of these parameters. The efficiency of emergence varies greatly for flux tubes with different geometric properties. A magnetic field with a larger initial field strength and radius is more efficiently transferred upwards. However, the magnetic field strength alone is not sufficient to predict if the magnetic field will emerge. A highly curved magnetic flux tube with a low twist emerges less efficiently than its counterpart with lower curvature and a similar twist. The efficiency of emergence increases with the twist in a highly curved magnetic flux tube.

Large-scale magnetic flux emergence from the convection zone creates δ-sunspots and sheared PILs, which are crucial for the understanding of flare eruptions and CMEs [3,4,16]. In [4], flux-emergence simulations were conducted on four types of ARs (spot-spot, spot satellite, quadrupole, and inter-AR), which were suspected to cause strong flares. Highly sheared PILs with a strong field and high gradient were created by the combined effects of the advection, stretching, and compression of magnetic fields.

In [16], a parameter study on the emergence of a twisted magnetic flux tube from the convection zone into the corona in a spot-spot-type AR was performed with a 3D MHD model. The initial twist of the magnetic flux tube was tuned to simulate the formation of δ-sunspots, under kink-stable, marginally stable, and unstable conditions, respectively.

In [17], the linear kink instability of isolated and twisted magnetic flux tubes was investigated by using a linearized MHD model. In [18], twisted magnetic flux tubes in an adiabatically stratified convection zone were simulated with a 3D MHD model. The development of helical kink instability in a uniformly twisted magnetic flux tube during its rise was studied.

In [19], the effects of field strength and twist on the emerging flux tubes were investigated. Self-similarity in the flux tube was observed when the magnetic field strength was changed, which did not occur when the twist was changed due to the complicated interaction between tension forces.

In [3], a density deficit was introduced at two spots in a quadrupole AR, along the length of a subphotospheric magnetic flux tube, to simulate the emergence of a twisted flux tube from the convection zone into a non-magnetized stratified atmosphere. The magnetic flux tubes emerged at two locations and formed a pair of bipoles in the photosphere. The two initially separated bipoles converged and formed a quadrupolar region, which contained a δ-shaped region between the two inner polarities. The magnetic field expanded outwards and formed two magnetic lobes, which later reconnected to form an envelope field to enclose successive emerging flux.

The sunspot number has been recorded over decades to better predict the solar activities, which significantly affect the Earth’s climate. Simulations of sunspot evolution under different conditions will gain more information about the underlying mechanisms. In this work, kink-stable, spot-spot-type δ-sunspots induced by flux emergence are simulated, under different subphotospheric initial conditions of magnetic field strength, radius, twist, and depth. Various plasma variables of the δ-sunspots are analyzed and compared with the observation data, including magnetic bipole structures, PILs, and temperature. The rest of this paper is organized as follows. The physical model and simulation scheme are presented in Section 2. The effects of the initial magnetic flux tube are simulated and discussed in Section 3. The time evolution of δ-sunspots is presented in Section 4, and some conclusions are drawn Section 5.

## 2. Physical Model and Simulation Scheme

**Figure 1:**
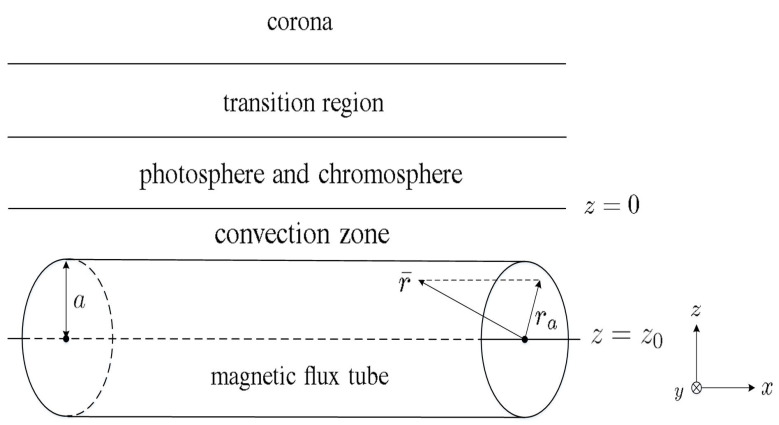
Simulation of the emergence of magnetic flux [12].

Figure 1 shows the simulation scenario for the emergence of magnetic flux. The initial stratification is composed of an adiabatic upper layer of the convection zone, a cool isothermal layer of the photosphere and chromosphere, a transition region, and a hot isothermal layer of the corona. A magnetic flux tube of radius *a* is initially located at $z=z_{0}$.

The MHD equations in cgsunits read:

(1) $$\begin{array}{ccc} & & \frac{\partial \rho }{\partial t}+\nabla \cdot \left(\rho \bar{v}\right)=0\\ & & \frac{\partial }{\partial t}\left(\rho \bar{v}\right)+\nabla \cdot \left(\rho \bar{v}\bar{v}\right)+\nabla p+\frac{1}{8\pi }\nabla B^{2}-\frac{1}{4\pi }\left(\bar{B}\cdot \nabla \right)\bar{B}=\rho \bar{g}\\ & & \frac{\partial E}{\partial t}+\nabla \cdot \left[\left(E+p_{t}\right)\bar{v}-\frac{\bar{B}}{4\pi }\left(\bar{v}\cdot \bar{B}\right)\right]=\frac{4\pi \eta_{e}}{c^{2}}{\left|\bar{J}\right|}^{2}+\rho \bar{v}\cdot \bar{g}\\ & & \frac{\partial \bar{B}}{\partial t}=\nabla \times \left(\bar{v}\times \bar{B}-\eta_{e}\bar{J}\right)\\ & & \bar{J}=\frac{c}{4\pi }\nabla \times \bar{B} \end{array}$$

where ρ is the mass density, $\bar{v}$ is the velocity, $\bar{B}$ is the magnetic field, $E=e+\rho |\bar{v}{|}^{2}/2+B^{2}/\left(8\pi \right)$ is the total energy density, *p* is the gas pressure, e=p/(γ−1) is the internal energy density, γ=5/3 is the ratio of specific heats, $\bar{g}$ is the gravitational acceleration, $\bar{J}$ is the current density, $p_{t}=p+B^{2}/\left(8\pi \right)$ is the total pressure, and $\eta_{e}=\frac{c^{2}}{4\pi \sigma_{e}}$ is the electric resistivity.

In this paper, the mass density, pressure, and temperature are normalized with respect to the values at z=0, which are $\rho_{p}=3\times 10^{-7}$$g/{\mathrm{cm}}^{3}$, $p_{p}=1.4\times 10^{5}$$\mathrm{dyn}/{\mathrm{cm}}^{2}$, and $T_{p}=5.6\times 10^{3}$ K. The normalization factors on length, time, speed, magnetic field, and acceleration are $L_{0}=170$ km, $t_{0}=25$ s, $v_{0}=\sqrt{p_{p}/\rho_{p}}=6.8$km/s (sound speed), $b_{0}=\sqrt{4\pi \rho_{p}v_{0}^{2}}=1.3\times 10^{3}$Gauss, and $g_{0}=v_{0}/t_{0}=2.7\times 10^{4}$$\mathrm{cm}/s^{2}$, respectively [12]. Thus, the MHD equations are normalized as:

(2) $$\begin{array}{ccc} & & \frac{\partial \rho^{\prime }}{\partial t^{\prime }}+\nabla \cdot \left(\rho^{\prime }{\bar{v}}^{\prime }\right)=0 \end{array}$$

(3) $$\begin{array}{ccc} & & \frac{\partial }{\partial t}\left(\rho^{\prime }{\bar{v}}^{\prime }\right)+\nabla \cdot \left(\rho^{\prime }{\bar{v}}^{\prime }{\bar{v}}^{\prime }\right)+\nabla p^{\prime }+\frac{1}{2}\nabla B^{\prime 2}-\nabla \cdot \left({\bar{B}}^{\prime }{\bar{B}}^{\prime }\right)=\rho^{\prime }{\bar{g}}^{\prime } \end{array}$$

(4) $$\begin{array}{ccc} & & \frac{\partial E^{\prime }}{\partial t^{\prime }}+\nabla \cdot \left[\left(E^{\prime }+p_{t}^{\prime }\right){\bar{v}}^{\prime }-{\bar{B}}^{\prime }\left({\bar{v}}^{\prime }\cdot {\bar{B}}^{\prime }\right)\right]=\eta {\left|{\bar{J}}^{\prime }\right|}^{2}+\rho {\bar{v}}^{\prime }\cdot {\bar{g}}^{\prime } \end{array}$$

(5) $$\begin{array}{ccc} & & \frac{\partial {\bar{B}}^{\prime }}{\partial t}=\nabla \times \left({\bar{v}}^{\prime }\times {\bar{B}}^{\prime }-\eta {\bar{J}}^{\prime }\right)\\ & & {\bar{J}}^{\prime }=\nabla \times {\bar{B}}^{\prime } \end{array}$$

where $E^{\prime }=e^{\prime }+\rho^{\prime }{\left|{\bar{v}}^{\prime }\right|}^{2}/2+B\prime {}^{2}/2$, $e^{\prime }=p^{\prime }/\left(\gamma -1\right)$, ${\bar{g}}^{\prime }=-\hat{z}g^{\prime }$, $g^{\prime }=1$, $p_{t}^{\prime }=p^{\prime }+B\prime {}^{2}/2$, and:

(6) $$\begin{array}{ccc} & & \eta =\left\{\begin{array}{cc} \eta_{0}\left(J^{\prime }/J_{c}-1\right),\; & J^{\prime }\geq J_{c},\rho^{\prime }<\rho_{c}\\ 0, & \mathrm{otherwise} \end{array}\right. \end{array}$$

is the anomalous resistivity model [4], with $\eta_{0}=0.1$, $J_{c}=0.1$, and $\rho_{c}=0.1$.

Table 1 lists four sets of subphotospheric initial conditions for the simulation and comparison of magnetic flux emergence. The parameters in Cases A, B/B’, and C were retrieved from [4,12,16], respectively. A magnetic flux tube is characterized by its magnetic field strength ($B_{0}^{\prime }$), twist (*q*), radius (*a*), depth ($z_{0}$), and the length of the buoyant part (λ). The corona is characterized by the height of the corona base ($z_{c}$), the temperature in the corona ($T_{c}$), plasma β, and a factor ϵ, which suppresses the emergence of flux tube ends. The parameter $\beta =p^{\prime }/\left(\right|\bar{B^{\prime }}{|}^{2}/2)$, the ratio between gas pressure and magnetic pressure, is specified at $z=z_{0}$. The minimum gird spacings are set to $\Delta x_{\min}$, $\Delta y_{\min}$ and $\Delta z_{\min}$.

The magnetic field strength and twist determine whether magnetic buoyancy instability will be triggered to erupt the magnetized plasma into the solar atmosphere [19]. The depth of the magnetic flux tube ($|z_{0}|$) determines the time it takes for the magnetic flux tube to reach the solar surface at z=0. The evolution of a δ-sunspot will be simulated and compared with the observation data under different subphotospheric initial conditions.

The initial temperature distribution in Case A is [12]:

(7) $$\begin{array}{ccc} & & T\left(z\right)=\left\{\begin{array}{cc} T_{p}-\frac{\gamma -1}{\gamma }z,\; & z<0\\ T_{p}, & 0\leq z<z_{t}\\ T_{c}^{\left(z-z_{t}\right)/\left(z_{c}-z_{t}\right)}, & z_{t}\leq z<z_{c}\\ T_{c}, & z\geq z_{c} \end{array}\right. \end{array}$$

where $T_{c}$ is the temperature in the corona, $z_{t}=10$ is the height of the transition-region base, and $z_{c}$ the height of the corona base. The initial temperature distribution in Cases B/B’ and C is [4,16],

(8) $$\begin{array}{ccc} & & T^{\prime }\left(z\right)=\left\{\begin{array}{cc} T_{p}-\frac{\gamma -1}{\gamma }z,\; & z<0\\ T_{p}+\frac{1}{2}\left(T_{c}-T_{p}\right)\left[\tanh\left(\frac{z-z_{c}}{w_{t}}\right)+1\right], & z\geq 0 \end{array}\right. \end{array}$$

where $w_{t}=2$ is the temperature scale height of the transition region.

The initial mass density and pressure are assumed to be in hydrodynamic equilibrium, satisfying $p^{\prime }=\rho^{\prime }T^{\prime }$. Given $T^{\prime }$, the variables $\rho^{\prime }$ and $p^{\prime }$ are computed numerically by solving $\partial p^{\prime }/\partial z=-g^{\prime }\rho^{\prime }$ in z≥0. In z<0, the mass density satisfies the adiabatic condition:

(9) $$\begin{array}{ccc} & & p\prime {}^{\left(1-\gamma \right)}T\prime {}^{\gamma }=\mathrm{const}. \end{array}$$

At t=0, a magnetic flux tube oriented in the *x*-direction appears in the convection zone at $z=z_{0}$, with [4]:

(10) $$\begin{array}{ccc} & & B_{x}^{\prime }\left(r_{a}\right)=B_{0}e^{-r_{a}^{2}/a^{2}}\\ & & B_{\theta }^{\prime }\left(r_{a}\right)=qr_{a}B_{0}e^{-r_{a}^{2}/a^{2}} \end{array}$$

where $B_{0}$ is the magnetic field strength, *a* is the radius of the tube, *q* determines the twist of magnetic field lines, and $r_{a}=\sqrt{y^{2}+{\left(z-z_{0}\right)}^{2}}$ is the radial distance to the tube axis. The azimuthal direction in the tube cross-section is labeled as the θ-direction. The axial field strength follows a Gaussian distribution in the radial direction. The azimuthal field endows the flux tube with a constant twist [3].

The plasma pressure inside the tube differs from that of the field-free atmosphere by $p_{1}\left(r_{a}\right)$, which satisfies the screw-pinch condition to balance the Lorentz force in the $r_{a}$-direction. The $p_{1}\left(r_{a}\right)$ factor satisfies [11]:

(11) $$\begin{array}{ccc} & & \frac{dp_{1}}{dr_{a}}=-\frac{d}{dr_{a}}\left(\frac{{B\prime }_{x}^{2}+{B\prime }_{\theta }^{2}}{2}\right)-\frac{{B\prime }_{\theta }^{2}}{r_{a}} \end{array}$$

By substituting (10) into (11) and integrating with respect to $r_{a}$, we obtain:

(12) $$\begin{array}{ccc} & & p_{1}=-\frac{{B\prime }_{x}^{2}+{B\prime }_{\theta }^{2}}{2}+\frac{a^{2}q^{2}B_{0}^{2}e^{-2r_{a}^{2}/a^{2}}}{4} \end{array}$$

To lift part of the flux tube towards the solar surface, a density deficit ($\rho_{1}$) is imposed along the flux tube as:

(13) $$\begin{array}{ccc} & & \rho_{1}\left(x,y,z\right)=\frac{p_{1}\left(r_{a}\right)}{p^{\prime }\left(z\right)}\rho \left(z\right)e^{-x^{2}/\lambda^{2}} \end{array}$$

where λ is the length of the buoyant tube.

In-house MATLAB code was developed to solve the MHD equations by using the HLLDRiemann solver with the Minmod slope limiter [20,21]. The constrained transport method was applied to maintain the divergence-free condition of the magnetic field [22]. Periodic boundary conditions were imposed in the x- and y-directions, and fixed boundary conditions were imposed in the upper and lower z boundaries. The minimum grid spacings, $\Delta x_{\min}$, $\Delta y_{\min}$, and $\Delta x_{\min}$, are listed in Table 1.

## 3. Effects of the Initial Magnetic Flux Tube

**Figure 2:**
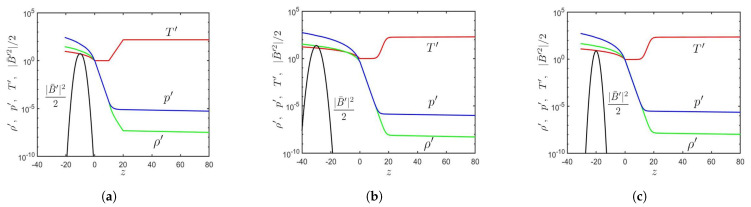
Initial conditions of $T^{\prime }$ (⎯⎯⎯), $|{\bar{B}}^{\prime }{|}^{2}/2$ (⎯⎯⎯), $\rho^{\prime }$ (⎯⎯⎯), and $p^{\prime }$ (⎯⎯⎯) along the *z*-axis: (**a**) Case A, (**b**) Case B, and (**c**) Case C.

Figure 2 shows the initial conditions of $\rho^{\prime }$, $p^{\prime }$, $T^{\prime }$, and $|B^{\prime }{|}^{2}/2$ along the *z*-axis, in Cases A, B, and C, respectively. In Case A, the initial values of $B_{0}=11.8/\sqrt{4\pi }$ and $z_{0}=-10$ [12] lead to $\beta =p^{\prime }/\left(\right|{\bar{B}}^{\prime }{|}^{2}/2)=10.1$, which is the lowest among these three cases. A δ-sunspot is expected, along with the eruption of both unmagnetized plasma and magnetized plasma. The magnetic flux tube has a shallower initial depth ($|z_{0}|$) than the other two cases, making it quicker to reach the solar surface (z=0).

In Case B, the initial values of $B_{0}=30/\sqrt{4\pi }$ and $z_{0}=-30$ [4] lead to β=17.2. A δ-sunspot is expected, and only unmagnetized plasma will be erupted. The magnetic flux tube has a deeper initial depth than the other two cases, taking the longest time to reach the solar surface.

In Case C, the initial values of $B_{0}=4.2$ and $z_{0}=-20$ [16] lead to β=30.1, which is the largest among these three cases. The magnetic flux tube is too weak to form an obvious sunspot.

**Figure 3:**
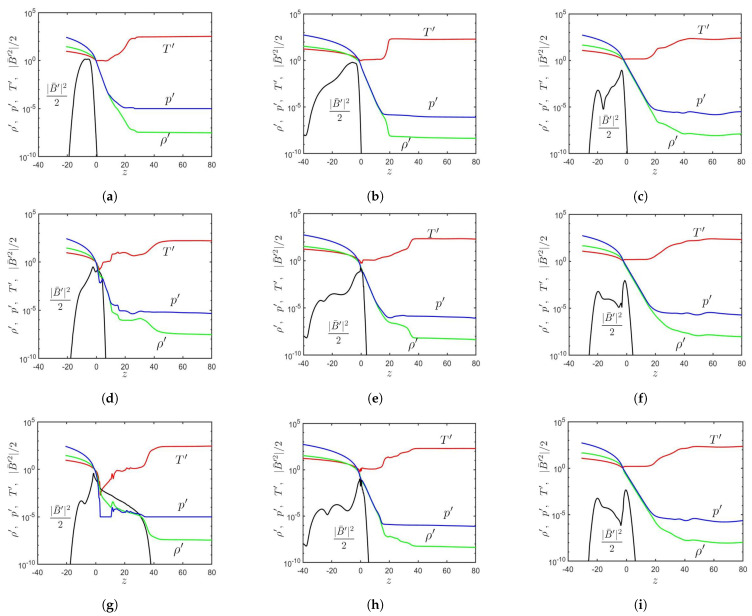
Distributions of $T^{\prime }$ (⎯⎯⎯), $|{\bar{B}}^{\prime }{|}^{2}/2$ (⎯⎯⎯), $\rho^{\prime }$ (⎯⎯⎯), and $p^{\prime }$ (⎯⎯⎯) along the *z*-axis: (**a**) Case A, $t^{\prime }=18.8$, (**b**) Case B, $t^{\prime }=118$, (**c**) Case C, $t^{\prime }=100$, (**d**) Case A, $t^{\prime }=48.8$, (**e**) Case B, $t^{\prime }=150$, (**f**) Case C, $t^{\prime }=131.95$, (**g**) Case A, $t^{\prime }=70.4$, (**h**) Case B, $t^{\prime }=169.97$, and (**i**) Case C, $t^{\prime }=150$.

Figure 3a–c show the distributions of $\rho^{\prime }$, $p^{\prime }$, $T^{\prime }$, and $|{\bar{B}}^{\prime }{|}^{2}/2$ in Cases A, B, and C, respectively, when the magnetic flux tube just reaches the solar surface (z=0). Figure 3d–f show these distributions about $\Delta t^{\prime }≃30$ after the tube reaches the solar surface; and Figure 3g–i show the distributions about $\Delta t^{\prime }≃20$ later.

In Case A, the rising magnetic flux tube pushes unmagnetized plasma upwards. It emerges in the lower solar atmosphere at $t^{\prime }=48.06$, pushing the plasma to z≃25. The plasma has higher $\rho^{\prime }$ and lower $T^{\prime }$ than its surroundings. At $t^{\prime }=70.4$, the magnetic flux tube carries magnetized plasma up to z≃40 by exerting magnetic buoyancy instability.

In Case B, the rising magnetic flux tube pushes unmagnetized plasma from the convection zone into the solar atmosphere. At $t^{\prime }=150$, the magnetic pressure reaches the peak around the solar surface and decreases rapidly in the *z*-direction. The magnetic flux tube staggers near the solar surface as magnetic buoyancy instability is not triggered. The situation remains the same at $t^{\prime }=169.97$.

In Case C, the magnetic flux tube reaches the solar atmosphere at about $t^{\prime }=100$. At $t^{\prime }=131.95$, the magnetic pressure peaks around the solar surface and decreases rapidly in the *z*-direction. At $t^{\prime }=150$, the magnetic flux tube is almost unchanged due to the weaker magnetic field compared with the other two cases.

**Figure 4:**
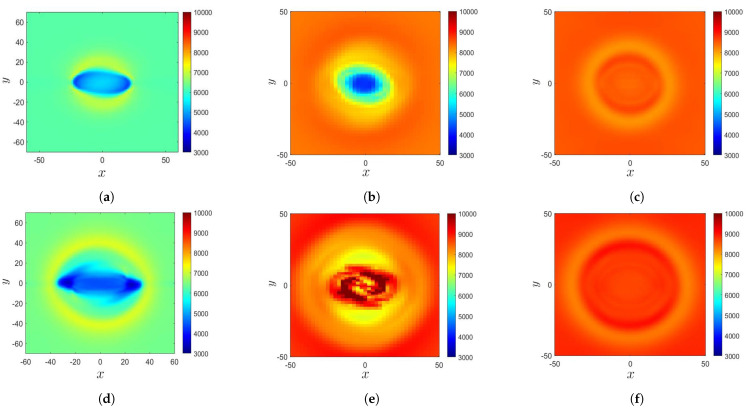
Evolution of the temperature distribution, *T* (K), on the solar surface (z=0): (**a**) Case A, $t^{\prime }=48.8$, (**b**) Case B, $t^{\prime }=150$, (**c**) Case C, $t^{\prime }=131.95$, (**d**) Case A, $t^{\prime }=70.4$, (**e**) Case B, $t^{\prime }=169.97$, and (**f**) Case C, $t^{\prime }=150$.

The sunspot begins to take shape when the magnetic flux tube reaches the solar surface. Figure 4a–c show the temperature distributions on the solar surface (z=0) in Cases A, B, and C, respectively, at the instants corresponding to the second row in Figure 3. Figure 4d–f show the temperature distributions on the solar surface in Cases A, B, and C, respectively, at the instants corresponding to the third row in Figure 3.

In Case A at $t^{\prime }=48.8$, a sunspot appears in the central region (−25<x<25) of the computational domain. The temperature in the central oval area is lower than its surroundings, with the lowest temperature of T≃4300 K. The oval area is enclosed by a ring area at higher temperature. Figure 4d shows that at $t^{\prime }=70.4$, the temperature in the sunspot decreases to T≃3300 K, and the oval area expands outwards.

In Case B at $t^{\prime }=150$, a sunspot appears in the central region, with the lowest temperature of T≃3500 K. The extent of the low-temperature area in the *x*-direction is shorter than that in Case A because the extent of the buoyant tube in Case A (λ=20) is larger than that in Case B (λ=8). A ring structure at a higher temperature encloses the central region. At $t^{\prime }=169.97$, the sunspot temperature increases, and its structure becomes fragmented. The temperature surrounding the fragmented area gradually increases to the background temperature. The sunspot area expands horizontally when the magnetic flux tube rises.

The plasma β at $z=z_{0}$ in Case C is higher than its counterparts in Cases A and B. In other words, the magnetic field in Case C is the weakest among these three cases. Figure 4c shows that at $t^{\prime }=131.95$, no significant temperature decrease is observed due to the weak magnetic field. A ring structure at a lower temperature encloses the central area. Figure 4f shows that at $t^{\prime }=150$, the background temperature keeps increasing, and the ring structure expands outwards.

**Figure 5:**
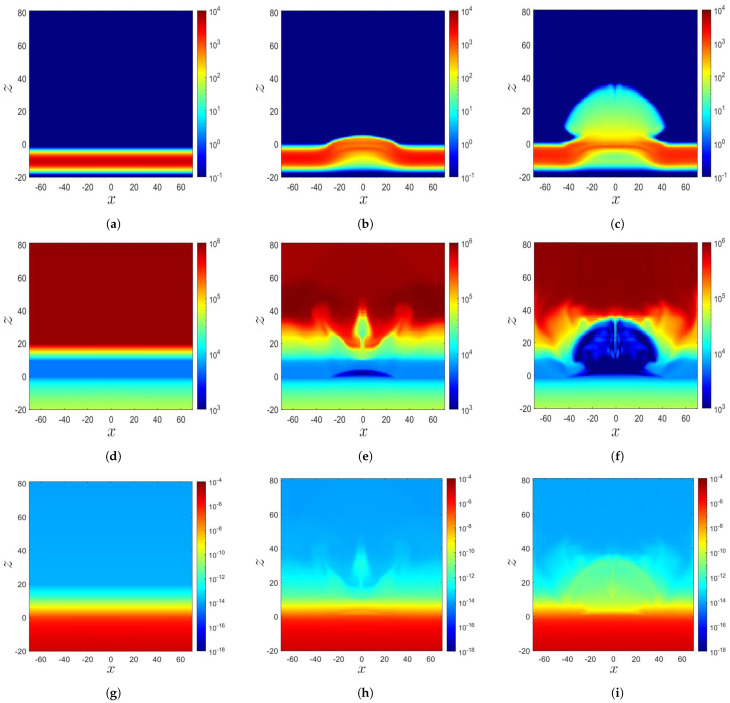
Distributions of the magnetic field, temperature, and mass density in Case A: (**a**) $|\bar{B}|$ (Gauss), $t^{\prime }=0$, (**b**) $|\bar{B}|$ (Gauss), $t^{\prime }=48.8$, (**c**) $|\bar{B}|$ (Gauss), $t^{\prime }=70.4$, (**d**) *T* (K), $t^{\prime }=0$, (**e**) *T* (K), $t^{\prime }=48.8$, (**f**) *T* (K), $t^{\prime }=70.4$, (**g**) ρ ($g/{\mathrm{cm}}^{3}$), $t^{\prime }=0$, (**h**) ρ ($g/{\mathrm{cm}}^{3}$), $t^{\prime }=48.8$, and (**i**) ρ ($g/{\mathrm{cm}}^{3}$), $t^{\prime }=70.4$.

**Figure 6:**
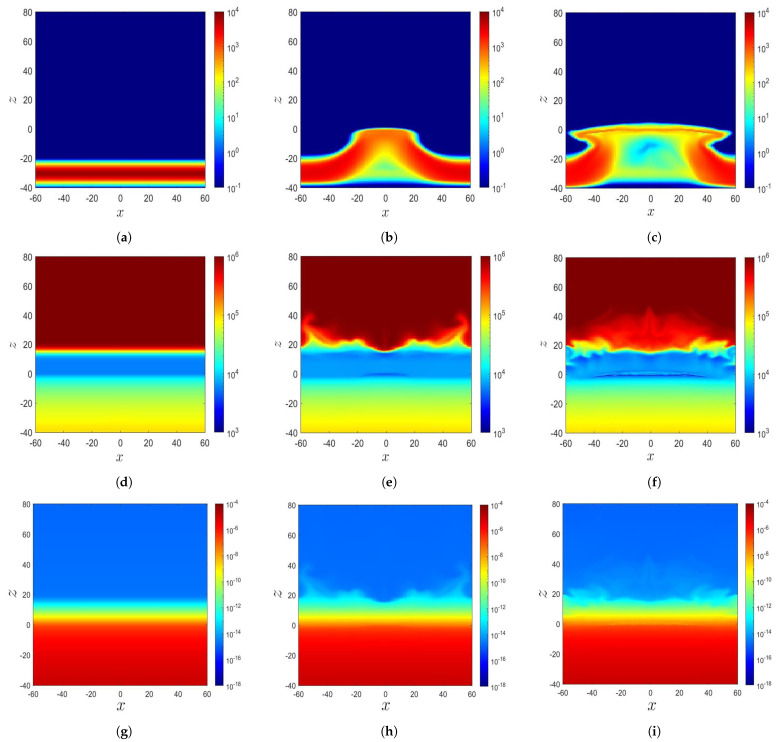
Distributions of the magnetic field, temperature, and mass density in Case B: (**a**) $|\bar{B}|$ (Gauss), $t^{\prime }=0$, (**b**) $|\bar{B}|$ (Gauss), $t^{\prime }=132.77$, (**c**) $|\bar{B}|$ (Gauss), $t^{\prime }=181.30$, (**d**) *T* (K), $t^{\prime }=0$, (**e**) *T* (K), $t^{\prime }=132.77$, (**f**) *T* (K), $t^{\prime }=181.30$, (**g**) ρ ($g/{\mathrm{cm}}^{3}$), $t^{\prime }=0$, (**h**) ρ ($g/{\mathrm{cm}}^{3}$), $t^{\prime }=132.77$, and (**i**) ρ ($g/{\mathrm{cm}}^{3}$), $t^{\prime }=181.30$.

When a magnetic flux tube rises, plasma is erupted from the convection zone to the solar atmosphere, causing a redistribution of the mass density and temperature in the photosphere and chromosphere (0≤z≤10), as shown in Figure 5 and Figure 6. Figure 5a shows that in Case A at $t^{\prime }=0$, a twisted magnetic flux tube is located at $z=z_{0}=-10$, with radius a=2.5. Figure 5d shows its initial temperature distribution depicted in (8), which is composed of an adiabatically stratified convection zone (z<0), a cool isothermal layer (0≤z<10) of the photosphere and chromosphere, a transition region (10≤z<20), and a hot isothermal layer (z≥20) of the corona. Figure 5g shows the mass density, which is computed from the temperature distribution $T^{\prime }$, by applying the hydrodynamic equilibrium condition $\partial p^{\prime }/\partial z=-g^{\prime }\rho^{\prime }$ in z≥0 and the adiabatic condition $p\prime {}^{\left(1-\gamma \right)}T\prime {}^{\gamma }=\mathrm{const}.$ in z<0.

Figure 5b shows that at $t^{\prime }=48.8$ (t=1220 s), a magnetic flux tube rises to the solar surface due to buoyancy. Then, magnetic buoyancy instability triggers a second expansion, pushing the magnetic flux tube with plasma to the solar atmosphere. Figure 5e shows that the temperature in the magnetized region is lower than that in the unmagnetized region. The unmagnetized plasma with T≃ 30,000 K is erupted from the convection zone (z<0) to the solar atmosphere. Figure 5h shows that the magnetized plasma has a higher ρ than its unmagnetized counterpart. The temperature distribution in the magnetized region protruding in the solar atmosphere changes significantly, because the solar atmosphere has a gas pressure several orders lower than that in the convection zone, making the former more sensitive to plasma eruption.

Figure 5c shows that at $t^{\prime }=70.4$ (t=1760 s), the second expansion continues, and the magnetic flux tube carrying plasma rises to z≃40. Figure 5f shows that the temperature drops significantly in the magnetized region. Figure 5i shows that the mass density erupts along with the magnetic flux and is about two orders of magnitude higher than that of the surrounding unmagnetized plasma. The magnetized plasma diffuses to the solar corona (z>20) and pushes the surrounding unmagnetized plasma outwards.

Figure 6a shows that in Case B at $t^{\prime }=0$, a twisted magnetic flux tube is located at $z=z_{0}=-30$, with radius a=3. Figure 6d shows that the initial temperature distribution depicted in (8) is composed of an adiabatically stratified convection zone (z<0), a cool isothermal layer (0≤z<10) of the photosphere and chromosphere, a transition region (10≤z<18), and a hot isothermal layer (z≥18) of the corona. Figure 6g shows the mass density distribution, which is derived from the temperature distribution by applying the hydrodynamic equilibrium $\partial p^{\prime }/\partial z=-g^{\prime }\rho^{\prime }$ in z≥0 and the adiabatic condition $p\prime {}^{\left(1-\gamma \right)}T\prime {}^{\gamma }=\mathrm{const}.$ in z<0.

Figure 6b shows that at $t^{\prime }=132.77$, the magnetic flux tube rises to the solar surface (z=0), driven by buoyancy due to the mass deficit. Figure 6e shows that when the unmagnetized plasma at a lower temperature is erupted from the convection zone to the solar atmosphere, the temperature distribution at z=0 and −15<x<15 becomes lower than its surroundings. Figure 6h shows that the unmagnetized plasma is erupted to the corona up to z≃40. Since both the gas-pressure gradient and magnetic-pressure gradient appear in (3), the rising tube tends to maintain a force balance with the gas pressure outside the tube. Thus, as the magnetic pressure ($|{\bar{B}\prime }^{2}|/2$) around the solar surface becomes higher than its surroundings, the gas pressure ($p^{\prime }$) thereof decreases, accompanied by a lower temperature because $T^{\prime }=p^{\prime }/\rho^{\prime }$.

Figure 6c shows that at $t^{\prime }=181.30$, the magnetic flux tube slows down its rising and expands horizontally when it reaches the solar surface. The magnetic flux accumulates near the solar surface, leading to a higher magnetic field strength thereof than its surroundings. According to the Schwarzschild criterion, the stellar medium is stable against convection if $-dT/dz<g/C_{p}$, where $C_{p}$ is the heat capacity at constant pressure. Since the photosphere is assumed isothermal (dT/dz=0), the Schwarzschild criterion is satisfied, and hence, the stratification in the photosphere is stable against convection [19]. Figure 6f shows that the low-temperature region on the solar surface expands horizontally to −40<x<40, correlated with the horizontal expansion of the magnetic flux tube. Figure 6i shows that the unmagnetized plasma, which carries the pressure and mass density, but not a magnetic field, is erupted to about z≃40.

For comparison, the distributions of Case A shown in Figure 5 demonstrate that the magnetic flux tube and the magnetized plasma erupt to the solar atmosphere because the magnetic buoyancy instability triggers the second expansion. On the other hand, the initial configuration of magnetic flux tube in Case B endows a low tension force and hence fails to trigger magnetic buoyancy instability to raise the tube to the solar atmosphere.

## 4. Time Evolution of δ-Sunspots

**Figure 7:**
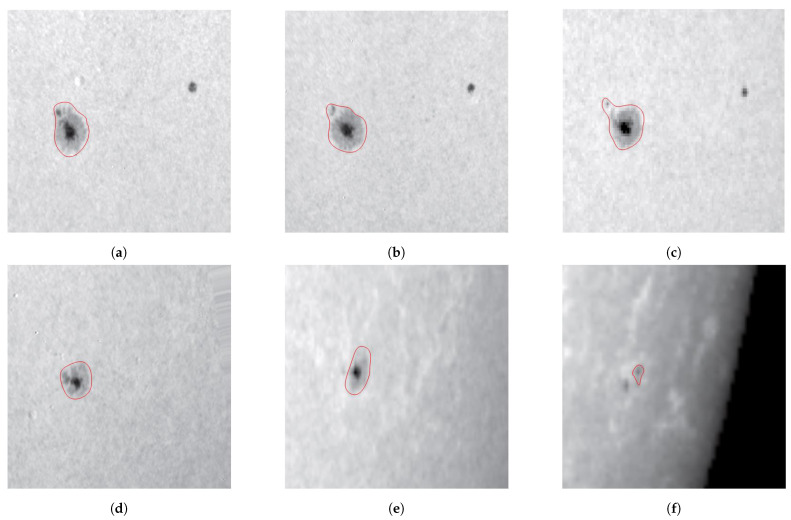
Evolution of NOAA Active Region (AR) 10773 [9] in white light observation, from 7 to 12 June 2005: (**a**) 7 June, (**b**) 8 June, (**c**) 9 June, (**d**) 10 June, (**e**) 11 June, and (**f**) 12 June. The field of view is $74^{\prime \prime }\times 74^{\prime \prime }$ or $5.38\times 10^{4}$ km by $5.38\times 10^{4}$ km. The spatial resolution is about $0.5^{\prime \prime }$ or 360 km.

Figure 7 shows a decaying follower sunspot, NOAA AR 10773, from 7–12 June 2005, with the spectroscopic observations in white light [9]. On 7 June, the sunspot showed a central umbra (the darkest part in the AR) and a surrounding penumbra (lighter than the central umbra). From 7–9 June, the penumbra decayed and shrank in size, but the umbra became darker than earlier. On 10 June, the umbra decayed, and the sunspot structure became blurred and fragmented. The temperature in part of the penumbra became close to the background temperature. Both the umbra and penumbra shrank in size and became fainter on 11 June. The umbra gradually contracted to a pore. On 12 June, the penumbra of the sunspot merged with the photosphere, and the temperature in the penumbra became almost the same as the background temperature.

**Figure 8:**
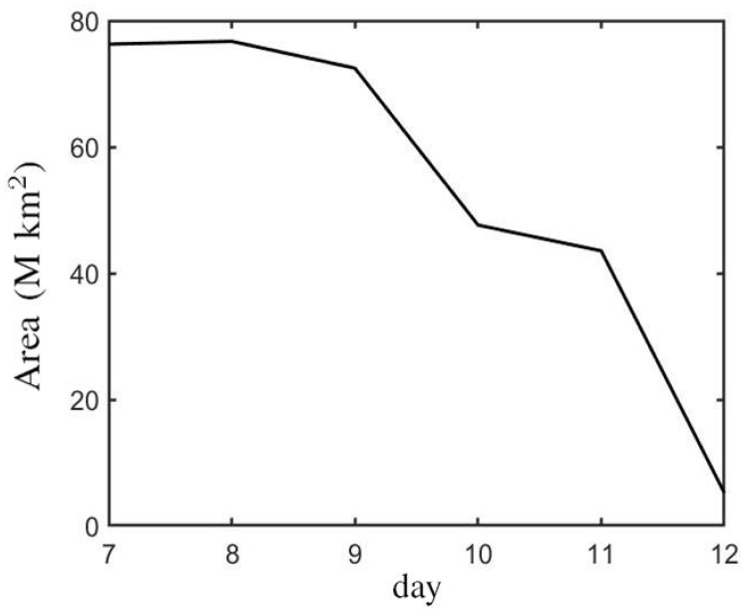
Time evolution of sunspot area of NOAA AR 10773 in white light observation, from 7 to 12 June 2005 [9].

Figure 8 shows the temporal variation of the sunspot area of NOAA AR 10773 in white light observation, from 7–12 June 2005 [9]. On 7 June, the sunspot area, encircled by a red contour in Figure 7, was $7.62\times 10^{7}$ km${}^{2}$. From 7–9 June, the sunspot area decreased from $7.62\times 10^{7}$ km${}^{2}$ to $7.24\times 10^{7}$ km${}^{2}$. On 10 June, the sunspot area dropped quickly to $4.76\times 10^{7}$ km${}^{2}$. On 11 June, the area of sunspot shrank to $4.35\times 10^{7}$ km${}^{2}$. On 12 June, the sunspot area reduced to $5.21\times 10^{6}$ km${}^{2}$. Note that the areas were estimated from the red contours encircling the sunspot, and the change of observation angle in Figure 7 was not taken into account.

**Figure 9:**
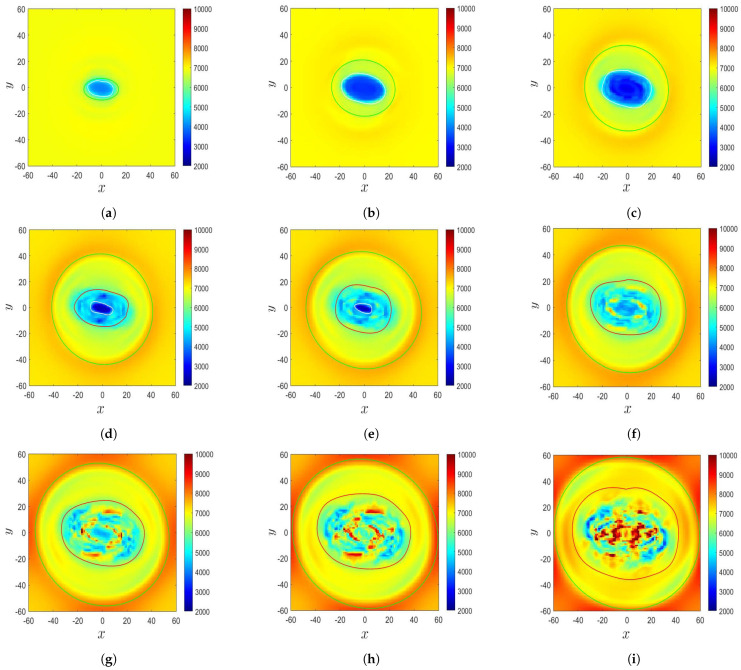
Evolution of the temperature distribution in the δ-sunspot in Case B: (**a**) $t^{\prime }=132.77$, (**b**) $t^{\prime }=141.87$, (**c**) $t^{\prime }=150.00$, (**d**) $t^{\prime }=156.43$, (**e**) $t^{\prime }=159.20$, (**f**) $t^{\prime }=163.87$, (**g**) $t^{\prime }=169.97$, (**h**) $t^{\prime }=174.39$, and (**i**) $t^{\prime }=181.30$. The area in each plot is 20,400 km × 20,400 km.

Figure 9 shows the simulated time evolution of the temperature distribution in the δ-sunspot of Case B, which displays some features similar to the observation in [9]. Figure 9a shows that at $t^{\prime }=132.77$ (t=3320 s), a δ-sunspot appears at the center, which is encircled by a green contour. The δ-sunspot contains an umbra of T≃4500 K, which is encircled by a white contour. The umbra is surrounded by a penumbra. Figure 9b shows that at $t^{\prime }=141.87$ (t=3546 s), The sunspot area and the umbra area expand horizontally. Figure 9c shows that at $t^{\prime }=150$ (t=3750 s), the temperature of the umbra decreases, with the lowest temperature dropping to T≃3500 K, and the brink of the umbra structure becomes blurred. The sunspot structure looks similar to that in Figure 7a. Figure 9d shows that at $t^{\prime }=156.43$ (t=3910 s), the δ-sunspot decays, and the sunspot structure becomes blurred. The umbra area begins to shrink, and a fragmented area appears surrounding the umbra, which is encircled by a red contour. Figure 9e ($t^{\prime }=159.20$, t=3980 s) and Figure 9f ($t^{\prime }=163.87$, t=4097 s) show that the temperature in a part of the fragmented area increases towards the background temperature. The sunspot becomes fragmented, and the umbra gradually disappears. A similar decaying process is observed in Figure 7c,d. Figure 9g ($t^{\prime }=169.97$, t=4250 s), Figure 9h ($t^{\prime }=174.39$, t=4360 s), and Figure 9i ($t^{\prime }=181.3$, t=4532 s) show that the temperature in the center of the sunspot keeps increasing, the sunspot structure becomes fainter, the fragmented structure becomes more complicated, the umbra contracts to a pore, and the temperature in a part of the fragmented area increases to T≃ 10,000 K. Similar features are observed in Figure 7e,f). Remember that as the magnetic flux tube rises, unmagnetized plasma is erupted from the convection zone into the solar atmosphere, and the temperature in the photosphere and chromosphere (0≤z≤10) increases. The background temperature of the photosphere keeps increasing because the eruption carries relatively hot plasma from the convection zone into the solar atmosphere, as shown in Figure 5 and Figure 6.

**Figure 10:**
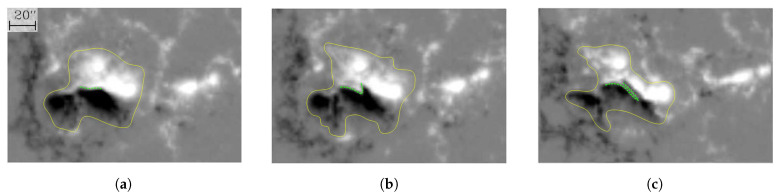
Magnetograms during the decay of the central δ-sunspot of NOAA AR 9026 [8]: (**a**) 2000-June-6 6:24:30 UT, (**b**) 2000-June-6 14:24:30 UT, and (**c**) 2000-June-7 14:24:36 UT. The PIL is marked by green dots, and the sunspot area is encircled by yellow contour. The length scale of $20^{\prime \prime }$ is about $1.45\times 10^{4}$ km.

Figure 10 shows the magnetograms during the decay of the central δ-sunspot of NOAA AR 9026 [8]. Figure 10a shows that at 6-June-2000 6:24:30 UT, a sheared polarity inversion line (PIL), marked by green dots, emerged on the solar surface. The sheared PIL appeared between two opposite magnetic polarities, with the light one being positive and the dark one being negative. The area of the δ-sunspot, encircled by a yellow contour, was about $1.48\times 10^{9}$ km${}^{2}$. Figure 10b shows that at 6-June-2000 14:24:30 UT, the sheared PIL rotated clockwise, and a switchback appeared when the two opposite magnetic polarities intruded into each other. The area of the δ-sunspot expanded slightly to $1.59\times 10^{9}$ km${}^{2}$. Figure 10c shows that at 7-June-2000 14:24:36 UT, the sheared PIL was elongated and folded back in the later stage of collapse, with the area of the δ-sunspot reduced to about $1.22\;\times \;10^{9}$ km${}^{2}$. In short, it took eight hours for the area of the sunspot to increase from $1.48\times 10^{9}$ km${}^{2}$ to $1.59\times 10^{9}$ km${}^{2}$, while the sheared PIL rotated clockwise and formed a switchback. Then, it took another 24 h for the PIL to elongate and fold back.

**Figure 11:**
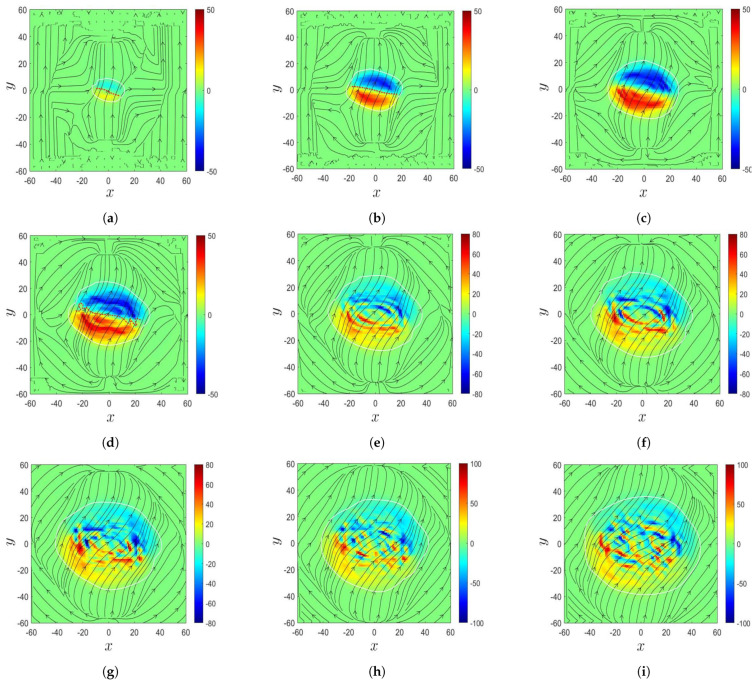
Evolution of magnetic field in the δ-sunspot in Case B: (**a**) $t^{\prime }=132.77$, (**b**) $t^{\prime }=141.87$, (**c**) $t^{\prime }=150.00$, (**d**) $t^{\prime }=154.54$, (**e**) $t^{\prime }=161.44$, (**f**) $t^{\prime }=166.50$, (**g**) $t^{\prime }=169.97$, (**h**) $t^{\prime }=174.39$, and (**i**) $t^{\prime }=181.30$. $B_{z}$ (Gauss) is in color; $B_{x}$ and $B_{y}$ are in arrows. The area in each plot is 20,400 km × 20,400 km.

Figure 11 shows the evolution of magnetic field in the δ-sunspot in Case B, to be compared with the observation in [8]. Figure 11a shows that at $t^{\prime }=132.77$ (t=3320 s), a magnetic bipole composed of two opposite magnetic polarities emerged on the solar surface, and a PIL, marked by red dots, appeared between these two opposite magnetic polarities. Similar features are observed in Figure 10a. Figure 11b shows that at $t^{\prime }=141.87$ (t=3546 s), the two opposite magnetic polarities expand, and the PIL is elongated. The magnetic field strength increases on the solar surface as the magnetic flux in the convection zone rises and accumulates near the solar surface. Figure 11c (at $t^{\prime }=150.00$, t=3750 s) and Figure 11d (at $t^{\prime }=154.54$, t=3864 s) show that each magnetic polarity splits into two fragments. The fragments share similar features to the switchback, and the PIL folds at its two ends, similar to the features observed in Figure 10b,c. Figure 11e (at $t^{\prime }=161.44$, t=4036 s), Figure 11f (at $t^{\prime }=166.50$, t=4162 s), and Figure 11g (at $t^{\prime }=169.97$, t=4250 s) show that the magnetic polarities intrude into each other, making the PIL difficult to identify. The magnetic polarities keep expanding, and the bipole structure transforms into multiple fragments. Figure 11h (at $t^{\prime }=174.39$, t=4360 s) and Figure 11i (at $t^{\prime }=181.30$, t=4532 s) show that the configuration of the magnetic polarities becomes more complicated, the magnetic field strength keeps increasing, and the fragments keep splitting.

**Figure 12:**
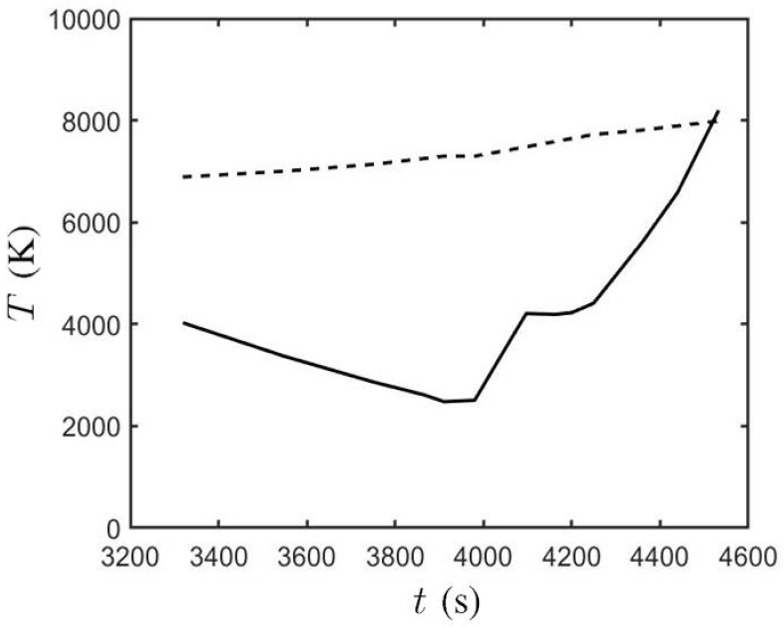
Temperature at sunspot center (⎯⎯⎯) and background (−−−) in Case B.

Figure 12 shows the temperature at the sunspot center and the background, respectively, in Case B, from $t^{\prime }=132.77$ to $t^{\prime }=181.30$. The sunspot temperature decreases at a constant rate from T≃4000 K at t=3320 s to T≃2400 K at t≃3900 s. Then, it increases to about 4200 K at t≃4100 s and reaches about 8000 K at $t^{\prime }=181.3$ (t=4532 s). The background temperature, which is estimated by taking the average of the temperature outside the green contour in Figure 9, increases linearly from T≃6890 K at $t^{\prime }=132.77$ (t=3320 s) to T≃7990 K at $t^{\prime }=181.30$ (t=4532 s). When the magnetic flux, accompanied by relatively hot plasma, is erupted from the convection zone into the solar atmosphere, the background temperature on the solar surface increases.

**Figure 13:**
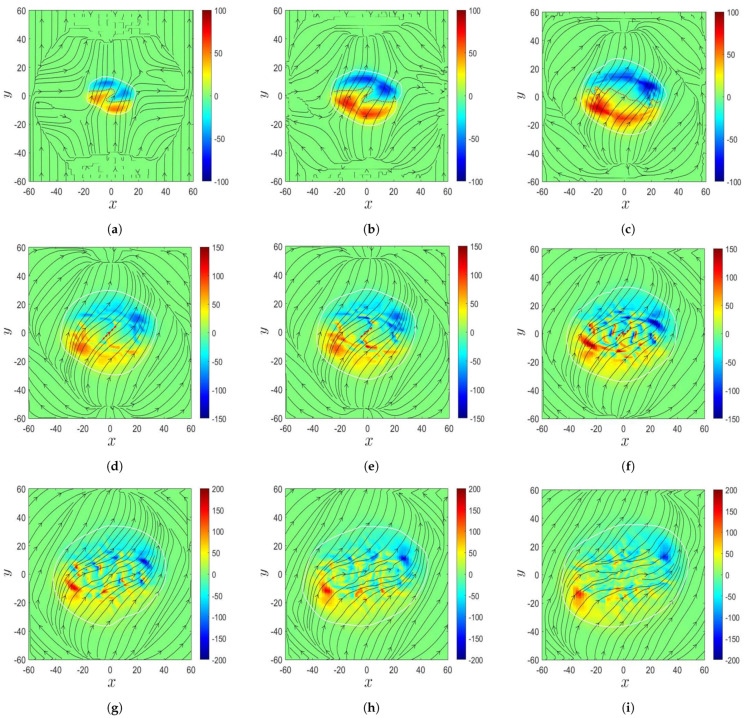
Evolution of magnetic field in the δ-sunspot in Case B’: (**a**) $t^{\prime }=132.77$, (**b**) $t^{\prime }=141.87$, (**c**) $t^{\prime }=150.00$, (**d**) $t^{\prime }=156.43$, (**e**) $t^{\prime }=159.20$, (**f**) $t^{\prime }=166.50$, (**g**) $t^{\prime }=169.97$, (**h**) $t^{\prime }=175.10$, and (**i**) $t^{\prime }=181.30$. $B_{z}$ (Gauss) is in color; $B_{x}$ and $B_{y}$ are in arrows. The area in each plot is 20,400 km × 20,400 km.

Figure 13 shows the evolution of magnetic field in the δ-sunspot in Case B’, which has the same physical parameters as in Case B, but at finer spatial resolution to confirm the phenomena of the fragments and switchback in Case B. Figure 13a shows that at $t^{\prime }=132.77$ (t=3320 s), a magnetic bipole composed of two opposite magnetic polarities emerges on the solar surface; and a PIL, marked by red dots, appears between the two opposite magnetic polarities. Both magnetic polarities display switchbacks, and the PIL takes a zig-zag shape. Figure 13b (at $t^{\prime }=141.87$, t=3546 s) and Figure 13c (at $t^{\prime }=150.00$, t=3750 s) show that the magnetic bipole expands in space, the PIL is elongated, and the magnetic field strength increases on the solar surface. The magnetic bipole structure becomes blurred, and the PIL is folded around its two ends. Figure 13d (at $t^{\prime }=156.43$, t=3910 s) and Figure 13e (at $t^{\prime }=159.20$, t=3980 s) show that each magnetic polarity splits into several fragments, and the original PIL becomes difficult to identify. However, a sheared PIL, marked by red dots, appears between two opposite fragments in the center of the δ-sunspot. The magnetic field strength keeps increasing. Figure 13f shows that at $t^{\prime }=166.50$ (t=4162 s), the sheared PIL turns into a zig-zag shape and the magnetic polarities keep splitting. Figure 13g (at $t^{\prime }=169.97$, t=4250 s), Figure 13h (at $t^{\prime }=175.10$, t=4378 s), and Figure 13i (at $t^{\prime }=181.30$, t=4532 s) show that the splitting of magnetic polarities becomes less active and the sheared PIL gradually disappears. The magnetic polarities keep expanding horizontally, and the magnetic field strength decreases.

Note that in Case B (with a relatively coarse grid), the numerical resistivity is relatively large, inducing larger numerical dissipation and causing the magnetic polarities to split into fragments. In Case B’ (with a relatively fine grid), the numerical dissipation is small. Thus, the magnetic polarities display switchbacks first and split into fragments later.

**Figure 14:**
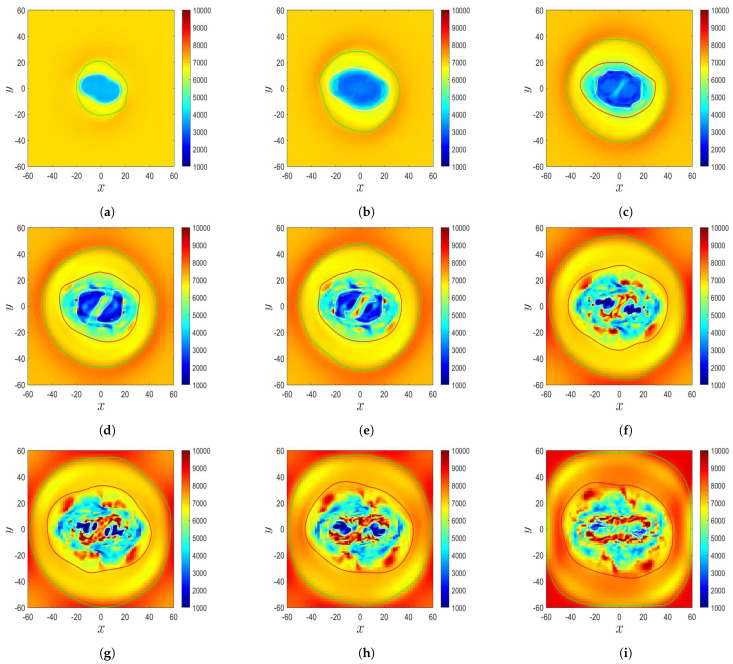
Evolution of temperature distribution in the δ-sunspot in Case B’: (**a**) $t^{\prime }=132.77$, (**b**) $t^{\prime }=141.87$, (**c**) $t^{\prime }=150.00$, (**d**) $t^{\prime }=156.43$, (**e**) $t^{\prime }=159.20$, (**f**) $t^{\prime }=166.50$, (**g**) $t^{\prime }=169.97$, (**h**) $t^{\prime }=175.10$, and (**i**) $t^{\prime }=181.30$. The area in each plot is 20,400 km × 20,400 km.

Figure 14 shows the evolution of the temperature distribution in the δ-sunspot in Case B’. Figure 14a (at $t^{\prime }=132.77$, t=3320 s) and Figure 14b (at $t^{\prime }=141.87$, t=3546 s) show that a δ-sunspot appears at the center, which is encircled by a green contour. The δ-sunspot contains an umbra of T≃4000 K, encircled by a white contour, and a surrounding penumbra. Both the sunspot area and the umbra area expand horizontally. Figure 14c shows that at $t^{\prime }=150$ (t=3750 s), the temperature of the umbra decreases to T≃3000 K, and the brink of the umbra structure becomes blurred. The temperature in the central part of the umbra increases, and the umbra begins to split. The temperature in a part of the penumbra increases and becomes fragmented, with the fragment area encircled by a red contour. Figure 14d shows that at $t^{\prime }=156.43$ (t=3910 s), the fragments in the penumbra keep splitting, and the sunspot structure becomes blurred. The umbra splits into two fragments, with a similar feature to that observed in [8]. In Case B, the split of the umbra is not well resolved due to the coarse horizontal grid. Figure 14e ($t^{\prime }=159.20$, t=3980 s) and Figure 14f ($t^{\prime }=166.50$, t=4163 s) show that the umbra area shrinks and the temperature in a part of the fragmented area increases towards the background temperature. The sunspot area and the fragmented area increase, and the fragment number keeps increasing. Figure 14g ($t^{\prime }=169.97$, t=4250 s), Figure 14h ($t^{\prime }=175.10$, t=4378 s), and Figure 14i ($t^{\prime }=181.3$, t=4532 s) show that the umbra area keeps shrinking and contracts to two pores. The sunspot area and the fragmented area keep expanding while the fragment number keeps increasing.

**Figure 15:**
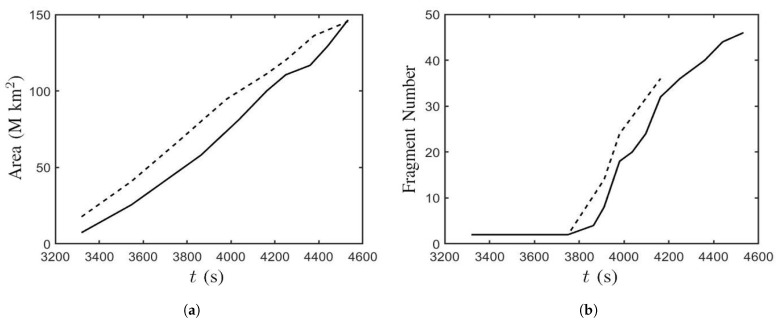
Time evolution of (**a**) the sunspot area and (**b**) the fragment number of the magnetic polarities; ⎯⎯⎯: Case B, - - - - - -: Case B’.

Figure 15 shows the time evolution of the sunspot area, encircled by white contours in Figure 11 and Figure 13, and the fragment number of the magnetic polarities in Cases B and B’, respectively. Figure 15a shows that the sunspot area increases linearly from $t^{\prime }=132.77$ (t=3320 s) to $t^{\prime }=181.30$ (t=4532 s), implying that the horizontal expansion rate of the magnetic flux tube on the solar surface is constant. In Case B, the sunspot area increases from $1.24\times 10^{7}$ km${}^{2}$ at $t^{\prime }=132.77$ (t=3320 s) to $1.28\times 10^{8}$ km${}^{2}$ at $t^{\prime }=181.30$ (t=4532 s). In Case B’, the sunspot area increases from $1.77\times 10^{7}$ km${}^{2}$ at $t^{\prime }=132.77$ (t=3320 s) to $1.45\times 10^{8}$ km${}^{2}$ at $t^{\prime }=181.30$ (t=4532 s). Figure 15b shows that at $t^{\prime }≃155$ (t=3875), the polarities begin to split and the bipole structure becomes fragmented. The fragment number increases to 32 at $t^{\prime }=166.50$ (t=4163) and 46 at $t^{\prime }=181.3$ (t=4532 s) in Case B. The fragment number increases to 36 at $t^{\prime }=166.50$ (t=4163) in Case B’. In Case B’, after $t^{\prime }=166.50$ (t=4163), the structure of the magnetic polarities becomes too blurred to count the fragment number.

**Figure 16:**
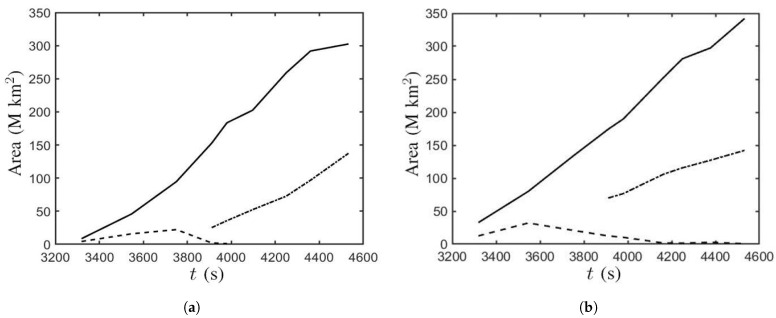
Time evolution of sunspot area (⎯⎯⎯), umbra area (- - - - - -), and fragmented area (−·−·−·−). (**a**) Case B and (**b**) Case B’.

Figure 16 shows the time evolution of the sunspot area, umbra area, and fragmented area, respectively. Figure 16a shows that the δ-sunspot in Case B appears at t≃3300 s and expands horizontally. The sunspot area increases linearly with time before t=4360 s, then the rate of increase becomes smaller. The umbra area increases along with the sunspot area. At t≃3750 s, the umbra area begins to decrease and eventually disappears at t≃4000 s. The fragmented area appears at t≃3900 s and increases in a similar manner to the sunspot area. Figure 16b shows that the δ-sunspot in Case B’ appears at t≃3300 s and expands linearly with time. The umbra area expands along with the sunspot area. The umbra area begins to decrease at t≃3550 s and reduces to 0.72 M km${}^{2}$ at $t^{\prime }=181.30$ (t=4532 s). The fragmented area appears at t≃3900 s and expands along with the sunspot area.

In summary, Figure 2 and Figure 3 show that the magnetic flux erupts into the solar atmosphere if magnetic buoyancy instability is triggered. Otherwise, it will accumulate near the solar surface. Figure 5 shows that the magnetic flux tube rises to the solar surface due to buoyancy. Then, magnetic buoyancy instability triggers a second expansion to push the magnetic flux tube into the solar atmosphere, carrying magnetized plasma. The temperature in the magnetized plasma is lower than that its surrounding unmagnetized region. The mass density of the former is about two orders of magnitude higher than that in the latter. The magnetized plasma diffuses into the solar corona and pushes its surrounding unmagnetized plasma outwards. Figure 6 shows that the rise of the magnetic flux tube slows down and expands horizontally when it reaches the solar surface. The magnetic flux tube fails to rise into the atmosphere because magnetic buoyancy instability is not triggered. Thus, the plasma erupted into the corona is unmagnetized. The temperature in the lower solar corona decreases when the plasma at lower temperature erupts from the convection zone.

Figure 10 shows two opposite magnetic polarities and a PIL on the solar surface, and a switchback was observed in the magnetogram during the decay phase of the central δ-sunspot of NOAA AR 9026. Figure 11 shows the simulated evolution of the magnetic field in the δ-sunspot. A magnetic bipole composed of two opposite magnetic polarities emerges on the solar surface, with a PIL appearing between them. The two magnetic polarities intrude each other, leading to a switchback in each polarity. As the magnetic bipole expands horizontally, the PIL is elongated and turns into a zig-zag shape. Figure 13 shows the evolution of magnetic field in the δ-sunspot at a finer spatial resolution. Each magnetic polarity splits into several fragments, and a sheared PIL appears in the center of the δ-sunspot.

Figure 7 shows a decaying follower sunspot, NOAA AR 10773. From 7 to 12 June 2005, the umbra and penumbra shrank in size and became fainter, with the umbra gradually contracting to a pore. Figure 9 shows that when the δ-sunspot decays, the umbra area shrinks and the brink of the umbra structure becomes blurred. The sunspot becomes fragmented, and the umbra finally contracts to a pore. Figure 14 shows that the temperature in the central umbra area increases and the umbra splits into two fragments, forming a sunspot pair. The temperature distribution in the penumbra becomes fragmented.

## 5. Conclusions

We simulated the time evolution of δ-sunspots induced by magnetic flux emergence, under different subphotospheric initial conditions of the magnetic flux tube, including the magnetic field strength, radius, twist, depth, and length of the buoyant part. A magnetic flux tube under low tension force does not trigger magnetic buoyancy instability and hence will not rise to the solar atmosphere. It slows down and expands horizontally when reaching the solar surface. The magnetic flux accumulates near the solar surface, with the plasma erupting to the corona without the magnetic field. If magnetic buoyancy instability is triggered, a second expansion will push the magnetic flux tube into the solar atmosphere, carrying magnetized plasma. The temperature in the erupted plasma is lower than its surrounding corona, and the mass density is about two orders of magnitude higher. The magnetized plasma diffuses into the solar corona and pushes its surrounding unmagnetized plasma outwards.

During plasma eruption, as simulated in Cases B and B’, a magnetic bipole composed of two opposite magnetic polarities emerges on the solar surface, with a PIL between the two polarities. The two polarities display switchbacks, similar to the observation in the central δ-sunspot of NOAA AR 9026. Each magnetic polarity splits into several fragments later, and a sheared PIL appears in the center of the δ-sunspot. The magnetic polarities in Case B split directly into fragments, while those in Case B’ display switchbacks and a zig-zag PIL first before splitting into fragments. An umbra area appears in the temperature distribution and expands along with the sunspot area. The umbra splits into two fragments, forming a sunspot pair. As the δ-sunspot decays, the umbra area shrinks, and its brink becomes blurred. The temperature distribution in the penumbra becomes fragmented. The simulated fragmentation phenomenon in some δ-sunspots may provide leads for future observations in the field.

## Figures and Tables

**Table 1:** Initial conditions of the subphotospheric magnetic flux tube.

	$B_{0}^{\prime }$	*q*	*a*	$z_{0}$	$z_{c}$	$T_{c}$	β	λ	ϵ	$\Delta x_{\min}$	$\Delta y_{\min}$	$\Delta z_{\min}$
Case A [12]	$11.8/\sqrt{4\pi }$	−0.4	2.5	−10	20	150	10.1	20	0	1	1	0.3
Case B [4]	$30/\sqrt{4\pi }$	−0.2	3	−30	18	150	17.2	8	0.2	1.2	1.2	0.25
Case B’ [4]	$30/\sqrt{4\pi }$	−0.2	3	−30	18	150	17.2	8	0	0.5	0.5	0.25
Case C [16]	4.2	−0.25	2	−20	18	200	30.1	8	0.2	1	1	0.3

$B_{0}^{\prime }$: magnetic field strength, *q*: twist, *a*: radius, $z_{0}$: depth, $z_{c}$: height of the corona base, $T_{c}$: temperature in the corona, $\beta =p^{\prime }/\left(\right|{\bar{B}}^{\prime }{|}^{2}/2)$ calculated at $z=z_{0}$, λ: length of the buoyant part, ϵ: the factor that suppresses the emergence of both tube ends.

## Data Availability

The study did not report any data.
